# Does anterior cruciate ligament reconstruction increase venous thromboembolism risk compared with knee meniscectomy under arthroscopy?

**DOI:** 10.1186/s12891-022-05216-w

**Published:** 2022-03-18

**Authors:** Long Pang, Pengcheng Li, Hui Li, Xin Tang, Jing Zhu

**Affiliations:** 1 Department of Orthopedics, Orthopedic Research Institute, West China Hospital, Sichuan University, Chengdu, 610041 China; 2 Department of Respiratory and Critical Care Medicine, West China Hospital, Sichuan University, Chengdu, 610041 China

**Keywords:** Arthroscopic knee surgery, Venous thrombosis, Anterior cruciate ligament reconstruction, Meniscectomy

## Abstract

**Background:**

This study compared the incidence of postoperative venous thromboembolism (VTE) between meniscectomy and anterior cruciate ligament reconstruction (ACLR) under arthroscopy and assessed whether ACLR increases the VTE risk compared with meniscectomy.

**Methods:**

A retrospective study of prospectively collected clinical data, including data on 436 patients ranging in age from 18 to 60 years who underwent ACLR or meniscectomy surgery, was performed between October 2018 and October 2019 in our hospital. All patients underwent routine VTE screening by venous ultrasonography in postoperative week 2 and then clinical follow-up at 4 and 6 weeks post-surgery. The incidence of VTE was calculated, and clinical factors such as age, sex, body mass index (BMI), smoking, concomitant procedure, Caprini score, and duration of tourniquet use were evaluated in relation to the risk factors for VTE.

**Results:**

A total of 320 patients who underwent arthroscopic ACLR or meniscectomy were available for analysis. Of these patients, 130 (40.6%) underwent ACLR, and 190 (59.4%) underwent meniscectomy. No cases of pulmonary embolism (PE) or femoral deep vein thrombosis (DVT) were reported in either group. Fourteen patients (10.8%) developed VTE in the ACLR group compared with 10 (5.3%) in the meniscectomy group, with no significant difference (*p* = 0.066). Among these patients, 4 (3.1%) patients in the ACL reconstruction group and 2 (1.1%) patients in the meniscectomy group had DVT confirmed by Doppler ultrasound (*p* > 0.05). ACLR, age, and BMI (OR = 3.129; 1.061; 1.435) tended to increase the risk of VTE, but the results were not statistically significant (*p* = 0.056, 0.059, 0.054).

**Conclusions:**

The incidence of VTE after ACLR and meniscectomy within 6 weeks post-surgery was 10.8 and 5.3%, respectively. ACLR, age, and BMI had a tendency to increase the risk of VTE.

## Background

Knee arthroscopy is performed for the treatment of many types of knee injuries, with over 5 million patients undergoing this procedure globally every year [1]. Knee arthroscopic surgery is minimally invasive and safe, with a relatively low risk of venous thromboembolism (VTE) [2]. A previous meta-analysis revealed an overall rate of deep vein thrombosis (DVT) of 9.9% [3]. Two recent studies with large populations reported DVT rates of 0.46% and pulmonary embolism (PE) rates from 0.05 to 0.11% [4, 5]. For most orthopaedic interventions, thromboprophylaxis can strongly reduce the risk of thrombosis, but it also increases the risk of bleeding [6, 7]. Therefore, whether prophylaxis should be used in patients undergoing arthroscopic surgery is still controversial.

Meniscus interventions and anterior cruciate ligament reconstruction (ACLR) are the two main procedures performed in knee arthroscopy. Although indications for meniscal repair are constantly expanding [8], meniscectomy is widely performed for meniscal injury to provide short-term symptomatic relief [9]. Normally, meniscectomy is simple, can be completed in a short time and has a lower risk of VTE. However, specific data on the risk of VTE after knee meniscectomy are limited. Compared with meniscectomy, arthroscopic anterior cruciate ligament (ACL) reconstruction, as the most important and popular surgical procedure in knee-related sports medicine, may lead to a higher risk of VTE due to the complexity of the ACLR procedure with tourniquet use and a relatively long surgical time [10–12]. One early study even showed that 41.2% of patients had DVT, as confirmed by magnetic resonance venography after ACLR [12]. However, subsequent studies reported inconsistent results following ACL reconstruction [13–19], and the value of thromboprophylaxis following ACL reconstruction is unclear [16, 18–22].

One recent study showed that different arthroscopy-related procedures may affect VTE risk after surgery [23]. There is a lack of consensus on how to weigh the different risks, and few studies have investigated risk factors specific for ACLR or meniscectomy [19]. Therefore, we performed a population-based, historical cohort study to estimate the incidence of VTE after arthroscopic meniscectomy (a simple knee surgery) and ACLR (a relatively complex surgery) and to specifically determine whether ACLR increases the VTE risk compared with knee meniscectomy. It is hypothesized that ALCR would lead to a higher risk of VTE than arthroscopic meniscectomy.

## Methods

### Study population

A retrospective study of prospectively collected clinical data including 436 patients, ranging in age from 18 to 60 years, undergoing ACLR or meniscectomy surgery was performed at our hospital from October 2018 to October 2019. Patients meeting one of the following criteria were excluded from the study: unwillingness to provide written informed consent; bilateral knee surgeries; concomitant other ligament reconstruction; multiple ligament reconstructions or repair; a history of DVT or PE; ongoing treatment with anticoagulant therapy; a history of cerebrovascular accident in the past 6 months; or severe renal or hepatic failure. Any other cause of immobility and cases with a follow-up less than 3 months were also excluded. All patients were routinely screened for VTE with compression venous duplex ultrasonography. Clinical data, including date of birth, sex, weight, height, and smoking, were recorded preoperatively.

### Surgical technique

According to the intervention procedures, the patients included were divided into two groups (the ACLR group and the meniscectomy group). In the ACLR group, all patients received primary ACL reconstruction, including single bundle and double bundle reconstruction; concomitant simple procedures, including simple debridement, partial meniscectomy, and simple meniscal repair (1 or 2 sutures), were permitted. In the meniscectomy group, all patients mainly received meniscectomy, including partial meniscectomy, subtotal meniscectomy and total meniscectomy; concomitant simple procedures, including simple debridement, removal of loose bodies, and chondroplasty, were also allowed. All surgeries were performed by the same team under general anaesthesia with a tourniquet inflated to 250 to 300 mmHg. No intra-articular drain was inserted in either group. All concomitant treatments, operative time, Caprini score [24] and tourniquet time were recorded.

### Postoperative management

No brace or knee immobilizer was used postoperatively for patients who underwent meniscectomy. Patients started full weight bearing and physiotherapy, including active motion exercises, ankle pump exercise and thigh muscle strengthening, on the first postoperative day. Patients could move but limited squat- and torsion-related movements with full loading within 6 weeks post-surgery.

For the patients who underwent single ACL reconstruction, no limitations were placed on motion when unweighted. Patients also started physiotherapy, including ankle pump exercise and thigh muscle strengthening, on the first postoperative day. If the patients underwent concomitant meniscal repair during the operation, their knees were limited to 0° of extension for 6 weeks when walking with partial weight bearing but were allowed knee flexion less than 90° without loading. After 6 weeks, these patients were encouraged to gradually begin walking normally.

A pain treatment protocol with nonsteroidal anti-inflammatory drugs (NSAIDs) was followed, and patients were allowed to use additional opioid drugs according to their pain scores. No anticoagulants were used. Physiotherapy was continued for 3 months. Patients were discharged from the hospital at 1 or 2 days postoperatively.

### Follow-up assessments

All patients routinely underwent follow-up clinical assessments at 2, 4 and 6 weeks after surgery and routinely received compression venous duplex ultrasonography before the operation and 2 weeks after surgery in the outpatient clinic. A standardized protocol for compression venous duplex ultrasonography was applied for both legs [25]. Patients were asked whether they had any clinical signs or symptoms of VTE postoperatively. At any other time, any patients found to have a clinical suspicion of VTE, including disproportionate levels of swelling, calf or thigh pain not consistent with the surgery, and/or respiratory symptoms, were investigated with appropriate imaging in the form of an ultrasound Doppler scan and/or computed tomography pulmonary angiogram at our hospital. Once radiologically confirmed as cases of VTE, appropriate anticoagulation therapy was commenced. Any patients found to have VTE were analysed to identify any possible risk factors, including surgical procedure, age, sex, body mass index (BMI), smoking, concomitant surgery, Caprini score and tourniquet time.

### Outcome measures

The primary measure was the incidence of major VTE, including DVT (total, proximal, and distal), PE, or both, up to 6 weeks post-discharge. Proximal DVT was defined as VTE of the popliteal or common femoral vein. Distal DVT was defined as VTE located in the distal part of the popliteal vein, including the tibial and peroneal veins. The secondary measures were the incidence of total VTE and odds ratios (ORs) for all independent predictors of VTE.

### Statistical analysis

The independent-samples t test was performed to compare outcomes with a normal distribution, and the Mann-Whitney U test was used when the data were not normally distributed. The chi-square and Fisher’s exact tests were used to compare categorical variables between the 2 treatment groups. Binary logistic regression was performed to identify significant independent predictors, and the resulting ORs with 95% confidence intervals (CIs) were calculated for all independent predictors of VTE and DVT for both groups. Statistical significance was set at *P* < 0.05 for all analyses, and all statistical analyses were performed using SPSS software version 22.0 (SPSS Inc., Chicago, IL, USA).

A power analysis was performed with PASS software version 15.0 (NCSS, LLC. Kaysville, Utah, USA) based on the acceptable level of significance, expected effect size, and underlying event rate of the population in previously published work. To detect a 10% difference between the two groups with 80% power and a *p* value of less than 0.05, 100 patients per group were required to reach a sufficiently narrow CI.

## Results

During the 1-year study period, 436 arthroscopic ACLR or meniscectomy procedures were performed at our institution. A total of 116 patients were excluded after applying the inclusion criteria. The remaining 320 patients who underwent arthroscopic ACLR or meniscectomy were available for analysis. The baseline characteristics and potential risk factors for these patients are listed in Table 1. There were 177 men (55.3%) and 143 women (44.7%), with a mean age of 34.8 ± 11.5. The average patient BMI was 23.7 ± 3.3 kg/m^2^. Of the patients, 130 (40.6%) received ACLR, and 190 (59.4%) received meniscectomy. There was no difference in BMI or smoking between the treatment groups, while there were significant differences regarding sex ratio, age, Caprini score, concomitant surgery, tourniquet time and operative time (*p* < 0.01). The detailed characteristics of the patients are shown in Table 1.

**Table 1:** Baseline characteristics of the patients

Characteristic	ACLR group (*n* = 130)	Meniscectomy group (*n* = 190)	*P* value
Male sex, n (%)	90(69.2)	87(45.8)	< 0.001
Age, y, mean ± SD	29.5 ± 8.5	38.5 ± 11.8	< 0.001
BMI, kg/m^2^, mean ± SD	24.0 ± 3.7	23.6 ± 3.0	0.315
Smoking, n (%)	20(15.4)	27(14.2)	0.771
Caprini score, mean ± SD	2.3 ± 0.6	2.7 ± 0.8	< 0.002
Concomitant surgery, n (%)	88(67.7)	57(30.0)	< 0.001
Tourniquet time, min, mean ± SD	61.6 ± 23.6	30.6 ± 15.6	< 0.001
Operative time, min, mean ± SD	76.6 ± 25.6	42.7 ± 17.8	< 0.001

*ACLR* anterior cruciate ligament reconstruction, *BMI* body mass index, *SD* standard deviation

### Incidence of VTE

No PE or femoral DVT was reported in either group. The overall incidence of major VTE was 6 of 320 patients (1.9%) within the 6-week follow-up, and 4 of the 6 patients (66.7%) were asymptomatic. DVT was detected in 5 cases at 2 weeks after surgery and in 1 case at week 4. Among the 6 patients who suffered from DVT, 4 (3.1%) patients in the ACL reconstruction group and 2 (1.1%) patients in the meniscectomy group had confirmed DVT (*p* > 0.05). In the 4 patients with DVT who underwent ACL reconstruction, all DVTs were detected in the distal DVT regions (3 in the posterior tibial veins, 1 in the peroneal vein). In the 2 patients with DVT who underwent meniscectomy, there was 1 popliteal DVT and 1 distal DVT (in the posterior tibial vein and peroneal vein).

The overall incidence of other VTE events was 18 of 320 patients (5.6%) within the 6-week follow-up. In the ACL reconstruction group, other VTE events in the venous calf of the plexus leg muscle were found in 10 (7.7%) patients, and other VTE events in the meniscectomy group were found in 8 (4.2%) patients by ultrasonography. No difference was found between the two groups (*p* = 0.184). In total, 14 and 10 patients developed VTE in the first and second cohorts, respectively, with no significant difference in incidence (10.8% vs. 5.3%; *p* = 0.066). The detailed results are shown in Table 2.

**Table 2:** The incidence of VTE after ACL reconstruction and meniscectomy

Outcome	ACLR group (*n* = 130)	Meniscectomy group (*n* = 190)	*P* value
Total VTE, n (%)	14(10.8)	10(5.3)	0.066
Major VTE, n (%)	4(3.1)	2(1.1)	0.373^a^
Proximal DVT, n (%)	0	1(0.5)	0.307^*^
Distal DVT, n (%)	4(3.1)	1(0.5)	0.178^a^
PE, n (%)	0	0	–
Other VTE, n (%)	10(7.7)	8(4.2)	0.184

*VTE* venous thromboembolism, *DVT* deep vein thrombosis, *PE* pulmonary embolism, *ACLR* anterior cruciate ligament reconstruction; * Fisher’s test; ^a^ Continuity correction test

### Risk factors for VTE

A binary multivariate logistic regression analysis was performed to evaluate the associations between VTE, DVT and clinical characteristics. ACLR (yes or no), sex (male or not), smoking (yes or no) and concomitant surgery (yes or no) were included as categorical variables, and age, BMI, Caprini score and tourniquet time were included as continuous variables. Surgical time was deleted before the logistic regression analysis due to its collinearity with tourniquet time (both tolerances: 0.18, VIF: 56.532, 57.134, respectively). The multivariate regression revealed that ACLR, age and BMI were associated, with ORs of 3.20 [CI 0.97–10.54], 1.06 [CI 1.00–1.13] and 1.15 [CI 1.00–1.30], respectively, with the development of total VTE, but the results were not statistically significant (*p* = 0.052, 0.059 and 0.054, respectively) (Table 3). No risk factor remained statistically significant for the development of major VTE during the study period (all *p* > 0.05).

**Table 3:** Multivariate logistic regression analysis of the associations between patient characteristics and VTE

Patient characteristics	Total VTE		Major VTE	
	*P* value	OR [95% CI]	*P* value	OR [95% CI]
ACLR vs. Meniscectomy	0.056	3.20 [0.97–10.54]	0.163	5.26 [0.51–54.30]
Age	0.059	1.06 [1.00–1.13]	0.310	1.07 [0.94–1.20]
Sex (male)	0.542	1.44 [0.51–3.66]	0.635	1.61 [0.22–11.60]
BMI	0.054	1.15 [1.00–1.30]	0.404	1.12 [0.86–1.44]
Smoking	0.248	0.44 [0.07–2.02]	0.955	0.93 [0.06–13.51]
Concomitant surgery	0.832	0.77 [0.36–2.30]	0.713	0.72 [0.12–4.24]
Caprini score	0.718	0.54 [0.48–2.87]	0.773	1.29 [0.23–7.07]
Tourniquet time	0.288	1.00 [0.99–1.03]	0.348	1.02 [0.98–1.06]

*ACLR* anterior cruciate ligament reconstruction, *VTE* venous thromboembolism, *OR* odds ratio, *BMI* body mass index

In the ACLR group, 88 patients (67.7%) had concomitant surgeries for associated meniscus and/or chondral injury. Among these patients, 8 presented with VTE, including 3 distal DVTs and 5 other VTEs. However, the logistic regression analysis revealed that concomitant minor procedures did not increase the risk of major VTEs (*p* = 0.904) or total VTEs (*p* = 0.222), while BMI was the only independent risk factor (*p* = 0.035, OR 1.19 [CI 1.10–1.40]) for developing VTE following ACL reconstruction (Table 4).

**Table 4:** Multivariate logistic regression analysis of the associations between patient characteristics and VTE in ACLR group

Patient characteristics	Total VTE		Major VTE	
	*P* value	OR [95% CI]	*P* value	OR [95% CI]
Age	0.213	1.06 [0.97–1.15]	0.567	1.04 [0.90–1.20]
Sex (male)	0.235	2.21 [0.60–8.13]	0.422	2.50 [0.27–23.46]
BMI	0.035	1.19 [1.10–1.40]	0.503	1.10 [0.83–1.46]
Smoking	0.630	0.55 [0.05–6.35]	0.998	0.00 [0.00–0.00]
Concomitant surgery	0.222	0.47 [0.14–1.58]	0.904	1.15 [0.11–12.03]
Caprini score	0.294	0.47 [0.11–1.94]	0.783	0.72 [0.07–7.35]
Tourniquet time	0.851	1.00 [0.98–1.03]	0.454	1.02 [0.97–1.06]

*ACLR* anterior cruciate ligament reconstruction, *VTE* venous thromboembolism, *OR* odds ratio, *BMI* body mass index

## Discussion

The principal findings of this study are that the incidence of total VTE was 10.8 and 5.3% after ACL reconstruction and meniscectomy, respectively, without a significant difference. The DVT rates determined using duplex ultrasound within 6 weeks after ACLR and meniscectomy were 3.1 and 1.1%, respectively. ACLR, age and BMI tended to increase the risk of VTE development; however, this tendency failed to reach statistical significance, and no risk factor was detected for major VTE. In the ACLR group, BMI was the only independent risk factor for VTE development following ACL reconstruction.

The reported incidence of VTE after ACLR varies in the existing literature. Oshiba H et al. [13] found that the incidence of DVT after ACL reconstruction detected by ultrasonography on postoperative day 7 was 6.3% (16/256). Among patients with DVT, the incidence of symptomatic DVT was 12.5% (2/16). Dong et al. [14] reported that the incidence of DVT among Chinese patients was 12.1% within 7 days of 282 ACL surgeries, and 55.9% (19/34) of the diagnosed DVT were symptomatic. Ye et al. [15] studied 171 patients with an overall DVT incidence of 14% by venography, but did not report the occurrence of symptomatic DVT. Marieke C et al. [16] conducted a prospective cohort study and found DVT happened to 13 patients. Among these patients, 69.2% (9/13) were asymptomatic proximal or distal DVT, whereas 30.8% (4/13) were symptomatic. Based on these findings, they suggested that prophylactic measures for DVT should be considered after arthroscopic ACLR to decrease the incidence of DVT, especially when risk factors are present [13–16], consistent with the guidelines of the French Society of Anaesthesia and Intensive Care [26].

However, several investigations with large cohorts of patients undergoing ACLR have reported inconsistent results. Jameson et al. [17] found the rate of symptomatic VTE to be approximately 0.44% (0.30% DVT and 0.18% PE) in 13,941 patients undergoing ACLR. Forlenza EM et al. [18] reported that the incidence of symptomatic VTE was 1.01 and 1.22% at 30 and 90 days, respectively, in 11,977 patients undergoing ACLR, as recorded in the Humana administrative claims database in Indiana. Gaskill et al. [20] reported that the incidence of symptomatic VTE was 0.53% among the 16,558 ACLR recorded in the Military Health Care System (MHS) database in the United States. Schmitz et al. [21] reported that the incidence of symptomatic VTE was 0.4% (0.34% DVT and 0.06% PE) in a cohort consisting of 26,014 primary and revision ACLR procedures obtained from the Swedish Knee Ligament Register in Sweden. These authors recommended against the routine use of thromboprophylaxis, which is in accordance with the American guidelines and recommendations of Swedish surgeons [19, 27].

In our study, all patients underwent duplex ultrasound scans postoperatively, which revealed an incidence of DVT after ACLR of 3.1%. This rate is close to the incidence found by Oshiba H et al. [13] and Marieke C et al. [16] but lower than that reported by Dong et al. [14] and Ye et al. [15]. This may be because 67.4% of the patients underwent reconstruction of the medial and lateral collateral ligament and posterior cruciate ligament at the same time in Dong et al.’s study [14]. In Ye et al.’s study, DVT was detected by venography, which may be more sensitive to the diagnosis of VTE than the ultrasound used in our study. In this present study, the incidence of symptomatic DVT among all DVT was 33.3% (2/6), which is consistent to those reported by some studies (12.5 to 55.9%) [13–16].

The reported incidence of symptomatic VTE or DVT/VTE (0.4 to 1.2%) in several large cohorts [17, 18, 20, 21] was significantly lower than that in these studies [13–16] and in our study, which can be mostly explained by the study design and whether screening of VTE was performed for all patients. The data for these large cohorts are generally obtained from local and national databases according to international classification of diseases (ICD) codes. Therefore, the accuracy of the information related to ICD codes should be questioned, as the incidence of asymptomatic VTE cannot be accurately recorded in these health care systems. Moreover, the data from local and national databases are usually reported by patients, and not all patients undergo VTE screening after surgery. In fact, higher rates of VTE are usually reported with the use of prospective diagnostic screening, which was performed in our study and several other studies [13–16] with a higher reported incidence of VTE.

Compared with ACLR, meniscectomy has been considered to have a lower risk of VTE because of its simplicity and its relatively shorter operative time. In one recently published review [28], major VTE (symptomatic VTE, DVT and PE) was reported in 4.75% of patients who received ACL reconstruction without prophylaxis and 0.72% of patients who received simple knee arthroscopic surgery (similar to meniscectomy), while the rate of VTE was 8.0% among patients who received ACLR and 1.96% among patients who underwent simple knee arthroscopic surgery, which is almost consistent with the result in our study. However, another investigation with a cohort of 12,595 patients reported conflicting results; specifically, the VTE rate after ligament reconstruction was 0.16%, while that after meniscectomy was 0.56% [29].

Age as a risk factor for VTE has been previously reported in several series of patients undergoing knee arthroscopic surgeries. Oshiba H et al. [13] found that patients aged ≥30 years have a higher risk of developing VTE. Ye et al. [15] reported that female patients and those aged 35 years were at a higher risk for VTE, and they recommended routine thromboprophylaxis in these patients. In several studies with large cohorts, Jameson et al. [17] found that age over 40 years was associated with an increased VTE risk. Forlenza EM et al. [18] reported risk factors including age ≥ 45, inpatient surgery, chronic obstructive pulmonary disease (COPD), tobacco use and concurrent posterior cruciate ligament (PCL) reconstruction, meniscal transplant and osteochondral allograft. Gaskill et al. [20] reported an increased odds of VTE in patients aged 35 years and over with a history of nicotine use, anticoagulant use, concomitant high tibial osteotomy (HTO), or concomitant PCL reconstruction. Schmitz et al. [21] reported that the only significant risk factor for VTE after surgery was age. In our study, logistic regression revealed that age has a tendency to increase the risk of developing VTE following knee arthroscopic surgery, although the difference failed to reach statistical significance. This may be because we excluded those patients who were older than 60 years in this study.

BMI is directly related to obesity and is usually considered an important part of the preoperative assessment. Increasing BMI has been shown to be associated with increased rates of unplanned hospital admissions and postoperative complications following arthroscopic procedures [30–32]. In one recently published study with 141,335 patients following arthroscopic surgery [33], the authors found that the most common complications were DVT (0.27%), and VTE risk factors included being overweight (25.0 ≤ BMI < 30, OR = 1.474) or diabetic with class I obesity (30.0 ≤ BMI < 35, OR = 1.469), which is partially consistent with the characteristics of the ACLR group in our study. In our study, BMI was associated with an OR of 1.151 for VTE (*p* = 0.054). A statistically significant difference would likely be obtained with an increase in the sample size in future studies.

Our study has some limitations. First, the retrospective design limited our assessment of the true effect of surgical intervention on the risk of VTE. Second, the number of ACLR patients was relatively small. The power analysis showed that a patient number of 100 per group was sufficient to reach a sufficiently narrow CI, but a study with a larger number of patients would increase its clinical importance. In our study, the tendency of ACLR, age and BMI to be associated with an increased risk of VTE was found, so a much larger patient group may strengthen the statistical significance. Third, the incidence of VTE in this study was mainly identified at 2 weeks after surgery by duplex ultrasound. DVT may develop in patients after they are discharged. All patients underwent follow-up for clinical assessments, but asymptomatic DVT may not have been detected. Therefore, the true overall incidence of DVT after ACL reconstruction is probably slightly higher than that shown in this study. Finally, contraceptive and NSAID use in our study by female patients was not recorded, and this may have an overall effect on the final incidence of DVT [22, 34].

## Conclusions

The incidence of VTE after ACL reconstruction and meniscectomy detected by duplex ultrasound within 6 weeks post-surgery was 10.8 and 5.3%, respectively. ACL reconstruction, older age and increased BMI tended to increase the risk of VTE following knee arthroscopic surgeries.

## Data Availability

All data generated or analysed during this study are included in this published article.

## Abbreviations

VTE: Venous thromboembolism
ACLR: Anterior cruciate ligament reconstruction
BMI: Body mass index
PE: Pulmonary embolism
DVT: Deep vein thrombosis
